# Long-Term Impact of a Medical School Course on the Intersection of Art and Medical History

**DOI:** 10.12688/mep.20112.1

**Published:** 2024-07-09

**Authors:** Bobbi G. Coller, Gabriel Slamovits, Barry S. Coller

**Affiliations:** 1 Department of Medical Education, Icahn School of Medicine at Mount Sinai, New York, NY, 10029, USA; 2 Icahn School of Medicine at Mount Sinai, New York, NY, 10029, USA; 3 Allen and Frances Adler Laboratory of Blood and Vascular Biology, The Rockefeller University, New York, New York, USA

**Keywords:** art history, medical history, wellness, humanities, medical education

## Abstract

**Background:**

One recent trend in medical education is the integration of humanities into the curriculum, including viewing works of art in museums, with analysis of short-term, but not long-term, impact. We developed a course for medical students, trainees, and faculty at the Icahn School of Medicine at Mount Sinai co-taught by an art historian and a physician/medical historian that features images of great works of art to make connections between art and medical history with the following goals: 1. To encourage the students to make careful and systematic observations, describe what they see to others in the group, and exchange their views respectfully, 2. To sensitize students to the patient’s experience of illness by discussing artists’ depictions of patients and the impact of their illness on family and friends, and 3. To highlight milestones in medical history by focusing on artworks that epitomize the state of medical care and science at a defined point in time. We have taught the course for more than a decade and so wanted to assess whether participating in the course had a long-term impact.

**Methods:**

We created and deployed a five-question survey to 167 students and received responses from 35 of those students.

**Results:**

97% of respondents answered that they still think about the course, and large majorities of the respondents indicated that the course, had an impact on how they viewed works of art (91%), their appreciation of the history of medicine (89%), and their observational skills (80%). More than half the students responded that the course sensitized them to the patient’s perspective of illness (63%) and had an impact on how they viewed their role as a physician (51%).

**Conclusions:**

Our course has had a long-term impact on the respondents across a wide range of professional and personal characteristics.

## Introduction

One of the major trends in medical education over the past two decades is the increasing integration of the humanities into the curriculum. Literature, music, and art have all been used to complement the intense scientific curriculum with the dual goals of developing important skills and providing a broad social context for understanding the role of the physician in society. Underlying these initiatives is the hope that these experiences will enhance the student’s compassion, empathy, and commitment to social justice ^1– 7^.

One of the more popular methods of melding the disciplines of humanities and medicine has been the introduction of works of art into the curriculum via visits to local museums in order to sharpen students’ observational skills, their ability to describe what they see, their ability to constructively exchange views with others in a group, and their ability to tolerate the uncertainty that is intrinsic to interpreting works of art. In these programs, works of art have primarily been chosen because they were available in the museum and because they depicted images that would facilitate achieving the objectives. The art historical context of the works is generally not considered or is incidentally introduced at the end of the session. Similarly, neither the medical historical significance nor connections to current medical practice of the work are generally considered.

A modest body of evidence has already been accumulated linking art observation training with attainment of some of these goals ^8– 16^. Dalia *et al.* performed a scoping review of the literature on published studies of the incorporation of art in the medical school and resident curricula ^17^. They narrowed their review to 28 studies in which the methodology was described and in which there was a postintervention assessment. They found wide variations in the structures of the programs and the assessment methodologies, and they concluded that incorporating art into medical education appears to improve visual perception skills, empathy, and personal reflection. They called for additional studies to define the optimum point of intervention during medical education, as well as follow-up studies to assess whether positive effects are lasting.

We sought to build on these programs by integrating our expertise in art history, in the history of medicine, and in medical education, by creating a course for medical students, postgraduate medical trainees, and faculty members based on in-depth discussions of works of art with importance in both art and medical history, as well as of works that would stimulate discussions of issues related to medicine and society. We created a series of seven sessions on a variety of topics ( Table 1) that are co-taught by B.G.C. and B.S.C. and we host guest lectures on a variety of related topics at the Icahn School of Medicine at Mount Sinai (ISMMS). The course also includes two visits to the Solomon R. Guggenheim Museum to view works from the permanent collection and current exhibits, and a visit to the Rare Book Reading Room of the New York Academy of Medicine to view great books in the history of medicine, geared to the course and the students’ interests extending back to the 16 ^th^ century. The visit to the New York Academy of Medicine is the final session each semester since the students invariably have a sense of awe seeing the authentic works we have discussed and placing them all in a historical context. We have taught the course each year since 2009, with the exception of 2020 because of the COVID-19 pandemic.

**Table 1.** Components of The Pulse of Art.

1. Sessions Taught by Bobbi and Barry Coller
- Observation in Medicine and Art
- Images of Illness
- Picturing Pandemic Diseases
- Skin Deep: Dermatology in Art
- Reading and Misreading Faces
- The Gross Clinic and the Ascendancy of American Science and Medicine in the 19 ^th^ Century
- The Vaccine Wars: Learning from Smallpox
2. Guest Lecturers (selections in different years depending on lecturer availability)
- Diane Brown, RxArt
- Anne Garner, Former Curator of Rare Books and Manuscripts, New York Academy of Medicine
- Rick Giudotti, Positive Exposure
- Hope Grayson, Painter
- Bert Hansen, Professor of the History of Science at Baruch College in New York
- Alexandra Horowitz, Adjunct Associate Professor at Barnard College
- Asti Hustvedt, Independent Scholar / Writer
- Joe Lovett, Lovett Stories and Strategies
- Arlene Shaner, New York Academy of Medicine Historical Collections Librarian
- Sharon Vatsky, Former Director School Programs, Solomon R. Guggenheim Museum
- Christina Yang, Former Director Adult Programs, Solomon R. Guggenheim Museum
3. Visits to Other Institutions
- The Solomon R. Guggenheim Museum
- The New York Academy of Medicine, Rare Book Reading Room

We set three primary goals: 1. To encourage the students to make careful and systematic observations, to describe what they see to others in the group, and to exchange their views respectfully, 2. To sensitize the students to the patient’s experience of illness by viewing and discussing artists’ depictions of sick individuals and the impact of their illness on those close to them, and 3. To highlight milestones in the broad sweep of medical history by focusing on artworks that epitomize the state of medical care and science at a defined point in time. Together, these lead to discussions of medical ethics and human compassion. Since we have been teaching the course for more than a decade to both students and faculty, and there is a gap in our understanding of any long-term effects of participating, we decided to assess whether participating in the course had a lasting impact as judged by responses to a survey focused on our primary goals.

Course description: The course is an elective in the ISMMS Academy for Medicine & the Humanities. It is offered in the fall semester as a series of weekly 90-minute sessions from September to December. Table 1 contains a list of the major components of the course, which includes: a series of sessions that two of us (B.G.C. and B.S.C.) jointly teach, guest lectures selected from those listed, visits to the Guggenheim Museum, which are led by members of the museum’s educational staff, and the final session at the New York Academy of Medicine Rare Book Reading Room led by the Rare Book librarian.

To provide a sense of the sessions that are co-taught by two of the authors, we provide descriptions of three of the sessions in Supplementary Materials covering the topics of *Observation in Medicine and Art; Skin Deep: Dermatology and Art;* and *The Gross Clinic and the Ascendancy of American Science and Medicine in the 19th Century*.

## Methods

This study was conducted under a protocol approved by the Institutional Review Board of the ISMMS (STUDY-20-01333) approved on February 2, 2021. Data collection began on July 9, 2021. All participants gave written informed consent to participate and were given the option of allowing or not allowing in writing each of their comments to be quoted anonymously.

Survey composition. The survey is reproduced in Table 2 and consists of questions to first determine: 1. whether the student took the course during the first five years or the second five years it was offered, and 2. whether they were a medical student, faculty member, or in another category when they took the course. This is followed by six yes/no questions related to: whether the students continue to think about the course; whether the course had an impact on their observational skills, how they view their role as a physician, how they view works of art, or their appreciation of the history of medicine; and finally, whether they felt that the course sensitized them to the patient’s perspective of illness. Students who answered yes to any of the questions were given an opportunity to add free text explaining their answer. If they entered any free text, they were then asked whether they gave permission for the text to be quoted anonymously in a publication.

**Table 2.** Survey questions.

When you took the Pulse of Art Course, were you a: Medical student, Faculty member, or Other?
Did you take the course before 2016 or in 2016 or thereafter?
Do you ever think about the course? If yes, what triggers your memory?
Do you think the course had an impact on your observational skills? If so, how?
Do you think the course had an effect on how you view your role as a physician? If so, how?
Do you think the course had an effect on how you view works of art? If so, how?
Do you think the course had an impact on your appreciation of the history of medicine? If so, how?
Do you think the course sensitized you to the patient’s perspective of illness? If so, how?

Survey deployment. We used the email addresses we had for the students when they took the course, augmented by contact information available from the ISMMS Office of Alumni Affairs. Together, this totaled 167 students, although not all participated in the entire course. Each student was sent an email signed by B.G.C. and B.S.C. inviting them to participate. Three additional email reminders were sent over the next year. Students who proceeded from the email to the survey were told the survey was anonymous. They were then asked if they gave their consent to participate, and if they did, they were given the survey to complete online. Their anonymous responses were entered into the Excel file.

### Data analysis

This was a descriptive study and thus the data were collected in a spreadsheet and the numbers of individuals who provided each response counted and recorded. These were converted into percentages of the entire group.

## Results

**Survey responses:** A total of 35 students provided completed surveys, and one student provided an incomplete survey that was excluded from the analysis. Table 3 presents the survey data, organized based on whether the student took the course during the first five years or the second five years it was offered, and whether the student was a medical student, physician, or in an Other category. Fifteen respondents were medical students when they took the course, 12 were ISMMS faculty members, and eight were in the Other category; the latter included two ISMMS non-physician staff members, one resident, one fellow, two physicians in other advanced postgraduate training programs, and two practicing physicians not affiliated with ISMMS. For ease of analysis, the Faculty and Other categories were combined into a single Physicians/Others category.

**Table 3.** Survey data during first five years or second five years offered.

Question	Students before 2016 [n; (%)]		Students 2016 or after [n; (%)]		Physicians/Others before 2016 [n; (%)]		Physicians/Others 2016 or after [n; (%)]		Total
	Yes	No	Yes	No	Yes	No	Yes	No	Yes
Do you ever think about the course?	4 (100%)	0	10 (91%)	1	12 (100%)	0	8 (100%)	0	34/35
Do you think the course had an impact on your observational skills?	3 (75%)	1	9 (82%)	2	10 (83%)	2	6 (75%)	2	28/35
Do you think the course had an effect on how you view your role as a physician?	1 (33%)	3	5 (45%)	6	8 (67%)	4	5 (63%)	3	18/35
Do you think the course had an effect on how you view works of art?	3 (75%)	1	10 (91%)	1	12 (100%)	0	7 (88%)	1	32/35
Do you think the course had an impact on your appreciation of the history of medicine?	3 (75%)	1	10 (91%)	1	10 (83%)	2	8 (100%)	0	31/35
Do you think the course sensitized you to the patient’s perspective of illness?	1 (75%)	3	7 (64%)	4	8 (67%)	4	5 (63%)	3	22/35

**Global analysis:** 97% (34/35) of respondents answered that they still think about the course. 80% or more of the respondents indicated that the course had an impact on how they viewed works of art (32/35; 91%), their appreciation of the history of medicine (31/35; 89%), and their observational skills (28/35; 80%). Smaller percentages, but still more than half the students responded that the course sensitized them to the patient’s perspective of illness (22/35; 63%) or had an impact on how they viewed their role as a physician (18/35; 51%). Table 4A– Table 4E contain selected free text comments made in response to each of the questions, categorized based on whether they were made by medical students or Physicians/Others.

**Table 4A.** Do you ever think about the course? / What triggers your memory?

**Medical Students**	[T]alking about art, when friends ask about the highlights of med school, when I talk to patients.
	When I visit art museums or exhibits, I often consider the work through a medical lens, which is something I never did before.
**Physicians/Others**	Thinking about how medicine and health is portrayed in art, specifically when I am at art museums.
	Whenever I bemoan the fact that the current education of our students is so narrow in its vision.
	Any time I think of medical history. Which is often.
	[W]henever I saw arts in hospital, especially during the moment I visited the ether dome at MGH.
	Whenever I am searching for art images, I remember Bobbi's detailed, comprehensive approach to a painting. I had never taken an art course previously and Bobbi's thorough approach of asking questions, i.e., dissecting a painting, as it were, while observing, is a fascinating one.

**Table 4B.** Do you think the course had an impact on your observational skills?

**Medical Students**	The course left in me an eagerness to ask questions about the things that we see - to pause and think about what is beyond the surface, and that is skill I will always carry with me.
	I think the approach to patients - considering how one may read an encounter as one reads a painting - was quite valuable.
	Yes, I feel as though this course helped me conceptualize medical observation as an art rather than as just a science. It helped me recognize the nuance that is involved in making keen medical observations.
	[P]erspective-taking; things can viewed from multiple angles, offering new methods/plans; discussion in large group settings can elucidate new points of view – every[one] observes things in a slightly different manner, additionally new perspectives can be incredibly impactful.
	Viewing a piece of art or a patient from different perspectives. Different perspectives enhances observational skills. You see things you otherwise would not have seen. This can translate into helping the patient in new ways.
	I see patients with more empathy and pay more attention to them as a whole person-focusing more on their body language and facial expressions
**Physicians/Others**	I think the course helped me pay closer attention to detail in my professional life and when looking at art. I do a lot of listening professionally in my work, and it has helped me augment my attentiveness to emotional cues.
	Looking at colors and lighting and facial expressions. It awakened my visual perception.
	Reminding myself to be more aware of art not just "formal art" but art in everyday life. To be more observant, and inclusive in my studies.
	I now look for pathologies in artwork, where I never did this before. I am more aware of how the artist's perceptions can be tainted by disease.
	It made me become a more careful and sensitive observer.
	I can better appreciate that my observational powers are limited by my experiences but can be broadened.

**Table 4C.** Do you think the course had an effect on how you view your role as a physician?

**Medical Student**	The course definitely presented a more humanistic view of medicine than our traditional medical school courses. By analyzing works done by patients or about patients, I felt that I had the opportunity to concretely see how physicians can have an impact on peoples' everyday lives.
**Physicians/Others**	I feel more grounded in history-- being able to see disease and the human experience from the eye of a medically trained professional even though it was art made by someone who is not medically trained.
	Seeing historical paintings of the changing styles and mores of the medical field gave me perspective and reflection. I think I am more aware of the human side of being a physician that is timeless in spite of differences in dress and posture.
	Enhanced appreciation of human beings.
	It is a wonderful course because it bridges the Arts and the Sciences/Medicine making it truly unique, giving a novel paradigm to what was CP Snow division of cultures. As physicians, we need to know rigorous science, proper diagnosis, analytical skills to evaluate study results of novel therapies but there is also a tremendous amount of human aspects of life/living, suffering and death and its metaphysical aspects. A bridged dialogue as this course offers is uniquely important for medical education.

**Table 4D.** Do you think the course had an effect on how you view works of art?

**Medical Students**	Art history has always been my side passion, my "coulda shoulda woulda" of sorts, we all have one of those other than medicine, so I think that when I heard this course was an offering I was so pleasantly overjoyed, it showed me that there was a way for me to not totally give up that passion down the line.
	This course has given me an appreciation for paintings and how much goes into making them.
	I find myself deliberately slowing down in museums and my own personal art collection to take in more of the work.
	Yes, I definitely consider art through a more medical lens now and I think this has really added depth to the way I approach art. It feels like solving a puzzle.
	The Collers did a really nice job demonstrating how their respective backgrounds inform the way they consume art, which made an impression on me!
	Art as lens to view the human condition and illness.
	There was a particular session on curating art for hospital settings. Through this session, I began to appreciate how art can foster a welcoming and less stressful environment in the hospital.
	It taught me to pay attention to details that I previously ignored
	I now take my time looking at each piece of artwork instead of rushing through a museum
**Physicians/Others**	Makes me think longer about the intentions of the artist and symbols that might be in the art work
	I view art in a systematic way, using the skills learned in the class to "take in" what I am appreciating, think why the artists did what they did, look for what may be missing, etc.
	I pay more attention to detail in art, want to know the historical context of the painting or art work, and am curious [about] the medical illness if depicted in the piece of art.
	It opened my eyes to painterly focus, emphasis and storytelling.
	Well you know the first second of looking at a patient sets the tone of the visit. The same when you approach art. [Y}ou see it first, [t]hen you analyze.
	I now see works that fall into two categories: art that depicts medical conditions (of the subjects), and art that is affected by medical conditions (of the artist).
	Absolutely--made me more aware of observing details within a painting, rather than looking at a work of art as a whole
	I actually have spent more time in exhibits [o]n each painting and looking into its historical aspects and temporal context in more detail since I attended this course.
	[P]ay more attention to the work of art and less time reading about the work.

**Table 4E.** Do you think the course had an impact on your appreciation of the history of medicine?

**Medical Students**	It made me want to learn more about the history of medicine.
	Certainly! I had never really given much thought to the history of medicine, but this course really highlighted just how much the history of medicine is intertwined with the history of art.
	I did not know much about the history of medicine prior to this course. Understanding the history of our day-to-day work can only further inform the present and our current roles/responsibilities. It is also humbling to know where we have come from
	The class was a fun exploration into the history of medicine, and made me want to read more.
	Learned about history of medicine from the lens of art - something I had not done before.
	I think interacting firsthand with the history of medicine and seeing books centuries old and learning from them was powerful. I went back to the NYAM [New York Academy of Medicine] after this experience as I was so inspired. I also loved learning about the architecture during our tour as well and started appreciating other similar forms around the city.
	Yes, it made me become more interested; recently, it has made me curious about better understanding the historical foundations of some racist aspects of the medical profession.
	Yes, even just exploring the rare book room with the Drs. Coller at the NYAM awoke in me an appreciation for how far medicine has come... and how even still, we know astonishingly little about how the human body works and how we can improve it.
	I appreciate the history of medicine to a greater extent.
**Physicians/Others**	I learned how much the history of medicine is relevant to how we practice medicine today.
	I am more curious on the history of certain illnesses and perceptions of illness. I think I had this curiosity before the course but that the course increased my curiosity.
	The images stay in my memory better than any historical text about history of medicine. But it also made me curious to read texts about history of art and medicine
	Absolutely--Barry's historical discussions were truly fascinating and detailed--covering some areas about which I was less familiar. The interface of Bobbi's discussions of the art with Barry's medical focus was superb and made the course exceptional.
	Absolutely! It is so important to understand the historical aspects of medical discoveries and societal aspects of illnesses and pandemics. This is truly highlighted in the course.
	The intersection of the arts and sciences has always been intriguing to me, especially through a historical lens. In 2021, history and historical context is now more important than ever in helping us understand the "why" of so much that is occurring.
	[V]ery humbling to think about the limited tools at the disposition of prior generations of physicians.
	It was fascinating how the history of medicine was documented through art. It is astonishing to see practices we now know to be ineffective, glorified on canvas.

**Subgroup analyses:** Comparing medical students to Physicians/Others: A total of 15 responses were from individuals who were medical students when they took the course compared to 20 who were in the Physician/Others group. Four of those in the medical student group took the course before 2016 and 11 took it after, compared with 12 and 8, respectively, in the Physicians/Others category.

87% (13/15) of the medical students responded that the course had an impact on how they view works of art compared to 95% (19/20) of the Physicians/Others.

87% (13/15) of the medical students responded that the course had an impact on their appreciation of the history of medicine compared to 90% (18/20) of the Physicians/Others.

80% (12/15) of the medical students responded that the course had an impact on their observational skills, the same percentage as the Physicians/Others (16/20).

53% (8/15) of the medical students responded that the course sensitized them to the patient’s perspective of illness compared to 65% (13/20) of the Physicians/Others.

40% (6/15) of the medical students responded that the course had an impact on how they viewed their role as a physician compared to 65% (13/20) of the Physicians/Others.

Comparing those who took the course before 2016 to those who took the course in 2016 or after, there were no obvious differences in the responses to the questions by medical students or Physicians/Others based on whether they took the course before 2016 or in 2016 or after ( Table 3).

## Discussion

One of the recognized limitations of studies of the impact of introducing the humanities in medical school curricula is the limited data on the long-term impact of such courses. Our data indicate that our course has had a long-lasting impact on at least a significant minority of participants, whether they were medical students, faculty, physicians in training, or staff members when they took the course. Our study is limited by the modest survey response rate and the likelihood that those who responded may have been motivated to respond because the course did have an impact on them. Other factors that may have contributed to the low response rate was its deployment by email to the last known email address of the students, the fact that our list included students who may not have attended more than a few sessions, and general survey fatigue by busy physicians. Medical students who took the course before 2016 had the lowest response rate, which may be attributed to the course having less of a lasting impact on them, their being in a phase of their career and family responsibilities where time is especially limited, and/or their contact information being less reliable because of the longer time since their graduation.

It is gratifying that 97% of those who did respond indicated that they still do think about the course and that their free text comments indicate that many of them feel that the course had a meaningful impact on how they think about and practice observation, and their appreciation of art and medical history. There was a clear gradient of impact, with very large majorities noting the impact on viewing art, appreciating medical history, and observation, a smaller majority noting the impact on being sensitized to the patient’s experience, and with a bare majority noting an impact on their role as a physician. In general, the responses were similar between those who took the course as medical students and those who were already physicians, and there were no obvious differences in responses based on whether the respondent took the course more than or fewer than 5 years before completing the survey.

The past decade has witnessed an increasingly intense focus on physician burnout and a recognition of the importance of promoting physician wellness ^18– 20^. The Action Collaborative on Clinician Well-Being and Resilience of the National Academy of Medicine (NAM) chose to address this need by sponsoring a traveling exhibit of 100 works of art produced by “artists of all skills and abilities to express what clinician burnout and clinician resilience/well-being looks, sounds, and feels like to them.” By recognizing the potential salutary effects of either making the art or viewing it as the exhibit traveled around the country, the NAM highlighted the power of both artistic expression and appreciation as contributors to physician wellness. He *et al.* noted the positive impact of their course, *The Art of Observation*, at the University of Texas Southwestern, on students’ feelings of burnout, with one student quoted as saying, “This class was rejuvenating for me. I think it’s a major reason why I did not go insane this semester. Art is so healing, and I think all of us should make more time to experience it.” Although we did not specifically ask a question related to physician wellness in the survey, we have performed surveys each year after each class and at the end of the course. One of the spontaneous comments from a student and another from a junior faculty member are very similar to that of the medical student quoted above.

‘…[Y]our course… was the bright spot of my week every week. It just happened to be right before our Wednesday clinical experiences where we go into the wards and practice interviewing/examining/interacting with real p[a]t[ient]s, and then debriefing via oral reports with our preceptors. These full-day sessions can be very exhausting, but I found having this course the night before really invigorating. Additionally, I always left the course feeling much more positive and motivated in general.’

‘The class gave me an "escape" from my regular work routine…It was the high-point at the end of my day, after long hours of patient care, resident education, and administrative duties.’

While there are many causes of physician burnout and proposed responses, cultivating an interest and appreciation of works of art can provide opportunities for intellectual growth and aesthetic enjoyment that can balance the increasing stresses on physicians. The stress of the COVID-19 pandemic on health care workers has resulted in many hospitals, medical schools, professional societies, local governments, and the World Health Organization establishing or expanding wellness programs that include the visual arts and partnerships with museums as an integral component ^21^. We hope that our course and others like it can make a contribution to this vital need and that future research can define the key elements that correlate with having a positive impact on medical student and health worker wellness.

## Ethics and consent

This study was conducted under a protocol approved by the Institutional Review Board of the Icahn School of Medicine at Mount Sinai (STUDY-20-01333) approved on February 2, 2021 . All participants gave written informed consent to participate and were given the option of allowing or not allowing in writing, each of their comments to be quoted anonymously.

## Data Availability

Zenodo: Long-Term Impact of a Medical School Course on the Intersection of Art and Medical History. https://zenodo.org/doi/10.5281/zenodo.10145559 ^22^. This project contains the following data: Coller - Long Term Impact Data.xlsx (Original data collected) Coller - Long Term Impact Supplementary Materials.pdf (Supplementary Materials) Data are available under the terms of the Creative Commons Attribution 4.0 International license (CC-BY 4.0).
