# Supplementary material for: Long-Term Impact of a Medical School Course on the Intersection of Art and Medical History
Source: MedEdPublish (2016). 2024 Aug 22;14:51. Originally published 2024 Jul 9. [Version 2] doi: 10.12688/mep.20112.2 (PMC11362722; doi:10.12688/mep.20112.2)
Supplement: Peer review history

# Reviewer report: Reviewer response for version 2

**DOI:** 10.21956/mep.22059.r38504

**Published:** 2024-10-17

**Authors:** Amanda Swain

**Affiliations:** 1 Department of Family Medicine & Community Health, University of Pennsylvania, Philadelphia, USA

I think authors addressed concerns though I think they still need an editor's eye on this copy to address run-on sentences and general readability.

Is the case presented with sufficient detail to be useful for teaching or other practitioners?

No

Is the work clearly and accurately presented and does it cite the current literature?

Partly

If applicable, is the statistical analysis and its interpretation appropriate?

Yes

Is the case’s background and context in sufficient detail?

Partly

Are all the source data underlying the results available to ensure full reproducibility?

Yes

Are the conclusions drawn adequately supported by the results?

Yes

Reviewer Expertise:

Faculty involved in medical humanities curriculum, elective planning and research.

I confirm that I have read this submission and believe that I have an appropriate level of expertise to confirm that it is of an acceptable scientific standard.

# Reviewer report: Reviewer response for version 2

**DOI:** 10.21956/mep.22059.r38506

**Published:** 2024-09-07

**Authors:** Ian Sabroe

**Affiliations:** 1 The University of Sheffield, Sheffield, England, UK

I am grateful for the responses to the review, which have appropriately discussed the comments/questions I raised.

Is the case presented with sufficient detail to be useful for teaching or other practitioners?

Yes

Is the work clearly and accurately presented and does it cite the current literature?

Partly

If applicable, is the statistical analysis and its interpretation appropriate?

Partly

Is the case’s background and context in sufficient detail?

Yes

Are all the source data underlying the results available to ensure full reproducibility?

Yes

Are the conclusions drawn adequately supported by the results?

Partly

Reviewer Expertise:

Medical humanities, biomedical science, respiratory medicine

I confirm that I have read this submission and believe that I have an appropriate level of expertise to confirm that it is of an acceptable scientific standard.

# Reviewer report: Reviewer response for version 2

**DOI:** 10.21956/mep.22059.r38505

**Published:** 2024-08-29

**Authors:** Vincenza Ferrara

**Affiliations:** 1 Sapienza University of Rome, Rome, Italy

I confirm that the paper is very important in the study of humanities in medical education and that is of an acceptable scientific standard to index.

Is the case presented with sufficient detail to be useful for teaching or other practitioners?

Partly

Is the work clearly and accurately presented and does it cite the current literature?

Partly

If applicable, is the statistical analysis and its interpretation appropriate?

Yes

Is the case’s background and context in sufficient detail?

Yes

Are all the source data underlying the results available to ensure full reproducibility?

Yes

Are the conclusions drawn adequately supported by the results?

Yes

Reviewer Expertise:

My area of ​​expertice and research is the application of the Visual Thinking Strategies method and other artistic practices for the training of medical and nursing students and continuing education in the healthcare area for developing skills and limiting stress of professionals.

I confirm that I have read this submission and believe that I have an appropriate level of expertise to confirm that it is of an acceptable scientific standard.

# Reviewer report: Reviewer response for version 1

**DOI:** 10.21956/mep.21537.r38111

**Published:** 2024-08-09

**Authors:** Amanda Swain

**Affiliations:** 1 Department of Family Medicine & Community Health, University of Pennsylvania, Philadelphia, USA

I really value attempting research on the longitudinal impact of medical humanities education. I agree with the authors that this is sorely needed.

**I have a few comments/suggestions below for the authors:**

 1. I recommend an editor review the piece. There are many run-on sentences that make sections hard to read. 

 2. The very first sentence is misleading. Medical humanities education is not a recent trend, it has been used in US medical education for 20+ years and much longer in European medical schools. I suggest this sentence is adjusted to reflect this fact.

 3. I think the questionnaire to alumni would have benefited from a Likert scale. The yes/no questions limit the utility of the information collected. I realize this data has already been collected, but in a methods section it would be helpful to discuss why they decided on yes/no vs something more detailed. I think this deficit could have been made up for by a content analysis of the answers (if there had been more of them).

 4. If the authors are going to comment on burnout it makes more sense to do so earlier in the article and then circle back to it in the discussion section. 

 5. Curious if the authors plan to use any of the data collected for this article to make changes to their current course.

Is the case presented with sufficient detail to be useful for teaching or other practitioners?

No

Is the work clearly and accurately presented and does it cite the current literature?

Partly

If applicable, is the statistical analysis and its interpretation appropriate?

Yes

Is the case’s background and context in sufficient detail?

Partly

Are all the source data underlying the results available to ensure full reproducibility?

Yes

Are the conclusions drawn adequately supported by the results?

Yes

Reviewer Expertise:

Faculty involved in medical humanities curriculum, elective planning and research.

I confirm that I have read this submission and believe that I have an appropriate level of expertise to confirm that it is of an acceptable scientific standard, however I have significant reservations, as outlined above.

# Response

**Published:** 2024-08-16

**Authors:** Barry Coller

**Affiliations:** Laboratory of Blood and Vascular Biology, Rockefeller University, New York, NY, USA

We thank Dr. Swain for the careful review and appreciate her identifying the need for data on the longitudinal impact of medical humanities as a strength of our manuscript. I have a few comments/suggestions below for the authors:

1. **Reviewer’s Comment:** I recommend an editor review the piece. There are many run-on sentences that make sections hard to read.
**Response: **We would be pleased to review any sentences that Dr. Swain or an editor thinks needs revision.
2. **Reviewer’s Comment: **The very first sentence is misleading. Medical humanities education is not a recent trend, it has been used in US medical education for 20+ years and much longer in European medical schools. I suggest this sentence is adjusted to reflect this fact.
**Response: **We thank Dr. Swain for the suggestion. We have modified the sentence accordingly. It now reads as indicated below.   Calls for integration of the humanities into medical education go back at least to the 1940s, but judging by PubMed citations, there has been a dramatic increase in interest and scholarship in this area during the past two decades.
3. **Reviewer’s Comment: ** I think the questionnaire to alumni would have benefited from a Likert scale. The yes/no questions limit the utility of the information collected. I realize this data has already been collected, but in a methods section it would be helpful to discuss why they decided on yes/no vs something more detailed. I think this deficit could have been made up for by a content analysis of the answers (if there had been more of them).
**Response: **Dr. Swain makes a good point. In reflecting on our choice of yes/no answers, we believe it derived from our sense that most of the answers to the questions would be binary, such as whether they still think about the course. We acknowledge that some of the others may have lent themselves to Likert scale assessment and will consider this for future research.
4. **Reviewer’s Comment: **If the authors are going to comment on burnout it makes more sense to do so earlier in the article and then circle back to it in the discussion section.
**Response: **We thank the reviewer for the suggestion, and we have now added the sentence below in the Introduction. Although not one of our stated goals, in the course of our analysis we noted that a number of respondent comments touched on the issue of physician burnout and wellness, a subject that has been a focus of recent attention, and so we briefly discuss the potential of courses like ours to contribute to physician wellness.
5. **Reviewer’s Comment: **Curious if the authors plan to use any of the data collected for this article to make changes to their current course.
**Response: **Based on the comments related to wellness, we are thinking about how to modify our course or create a related course that focuses more on this important issue. We now plan to ask specifically about wellness in our course survey.

# Reviewer report: Reviewer response for version 1

**DOI:** 10.21956/mep.21537.r38112

**Published:** 2024-08-09

**Authors:** Ian Sabroe

**Affiliations:** 1 The University of Sheffield, Sheffield, England, UK

I reviewed the paper of Coller et al with interest. The authors have been running a longstanding elective course which they describe as examining the intersection of art and medical history, with clear goals identified around observation skills, respectful discussion, the experience of illness, and understanding of medical history. To their credit, they wished to evaluate the impact of this course and have done so by means of a questionnaire.

 I applaud the desire to evaluate their course, and am interested to read the results. I wish the authors had not used leading questions in their survey to the extent that they have. Although the questions are open and inviting comment, they do have a leading nature by directing the respondents to consider the course and then their observational skills, physician role, etc. The questions could have focused more on lasting aspects without tying them to specific parameters, in order to generate a more open, unstructured reflection.

 I am also concerned about one line in the methods, where ‘students who answered yes’ to a question were invited to add free text to explain their answers. If people answered ‘no’, their reflections on the course and question would be equally valid and useful or important to capture.

 Regarding the data generated, as the authors note the numbers of responses are relatively small. Surveys were sent to 167 students (which would appear to be all/most of those taking the course over 10 years) and responses were from 35 students. The limitations of this small sample size are clearly acknowledged, and the limitations of the study appropriately discussed.

 Of those who responded, there are warm responses towards the aims of the course and positive recollections of it presented. (I note again that the survey did have somewhat leading questions, which provide a potential bias.) A series of ‘selected quotes’ are presented, without being clear how such quotes were chosen. Whilst it’s clear that the course had a very positive impact on some respondents, there’s a risk of selection bias in the choice of quotes presented in the main paper.

 The discussion of the data generated acknowledge the positive results gained and some of the limitations of the study. It would perhaps also be useful to set the discussion of this course a bit more in the overall offering of the School, and the importance it attaches to humanities-based options. Students who took this module may have also been influenced in how they viewed this particular module by participation in other courses, e.g. in narrative medicine. It’s too late to explore whether there were any mutual interdependencies/reinforcements between courses in the survey, but briefly discussing the School’s overall humanities offerings and attitudes to them might add value.

 A minor point, but in the supplemental materials, one link (reading and misreading faces) needs a proper link direct to the source material.

 As a point of discussion, the final paragraph of the discussion causes me to wonder how we present the importance and benefit of such courses. I cannot conceive of a future in which the practice of medicine will be easy. It will always be challenging, acutely and over a lifetime. I am all in favour of all wellness programmes. I support approaches that can positively impact on the lifetime stress of our career, as well as those that offer diverse ways of learning about illness. I think we should be incorporating humanities-based learning, knowledge and wisdom into training and practice as part of a standard to support clinician learning, practice, identity formation and wellbeing, for the benefits of our patients, societies, and ourselves. But… I believe the work of addressing clinician distress and burn-out requires deep reforms of working practice, clinician support etc, and that the work of creating more sustainable, survivable careers is to some degree an independent (and great) problem.

Is the case presented with sufficient detail to be useful for teaching or other practitioners?

Yes

Is the work clearly and accurately presented and does it cite the current literature?

Partly

If applicable, is the statistical analysis and its interpretation appropriate?

Partly

Is the case’s background and context in sufficient detail?

Yes

Are all the source data underlying the results available to ensure full reproducibility?

Yes

Are the conclusions drawn adequately supported by the results?

Partly

Reviewer Expertise:

Medical humanities, biomedical science, respiratory medicine

I confirm that I have read this submission and believe that I have an appropriate level of expertise to confirm that it is of an acceptable scientific standard, however I have significant reservations, as outlined above.

# Response

**Published:** 2024-08-16

**Authors:** Barry Coller

**Affiliations:** Laboratory of Blood and Vascular Biology, Rockefeller University, New York, NY, USA

We thank Dr. Sabroe for the review and for supporting our desire to evaluate the impact of our course. I applaud the desire to evaluate their course, and am interested to read the results.

**Reviewer’s comment:** I wish the authors had not used leading questions in their survey to the extent that they have. Although the questions are open and inviting comment, they do have a leading nature by directing the respondents to consider the course and then their observational skills, physician role, etc. The questions could have focused more on lasting aspects without tying them to specific parameters, in order to generate a more open, unstructured reflection.

**Response:** We did not intend for our questions to be “leading,” and we appreciate Dr. Sabroe’s acknowledging that the questions are “open and inviting comment.” Dr. Sabroe suggests that we could have focused more on “lasting effects without tying them to specific parameters in order to generate more open, unstructured reflection.” We agree that an open request for “lasting effects” would allow for broad reflection, but we were specifically interested in knowing whether we achieved our goals and so we tried to obtain this information by asking questions that we thought were presented in a neutral fashion. We balanced this by providing respondents an opportunity via open text to express unstructured thoughts and reflections. We also were concerned that fully unstructured questions would present significant analytical challenges.  

**Reviewer’s comment: **I am also concerned about one line in the methods, where ‘students who answered yes’ to a question were invited to add free text to explain their answers. If people answered ‘no’, their reflections on the course and question would be equally valid and useful or important to capture.

**Response:** We appreciate Dr. Sabroe’s comment, but we didn’t think that, for example, people who do not still think about the course would be interested in explaining why they no longer think about it. In addition, we did provide an opportunity for free text about anything they would like to add.

 Regarding the data generated, as the authors note the numbers of responses are relatively small. Surveys were sent to 167 students (which would appear to be all/most of those taking the course over 10 years) and responses were from 35 students. The limitations of this small sample size are clearly acknowledged, and the limitations of the study appropriately discussed. We, of course, would also have preferred a high response rate, but we felt that the 35 responses provided sufficiently valuable information to justify reporting our results. We appreciate Dr. Sabroe’s acknowledgement that we were transparent in reporting the response rate and noting the limitations of our study.

**Reviewer’s comment: **Of those who responded, there are warm responses towards the aims of the course and positive recollections of it presented. (I note again that the survey did have somewhat leading questions, which provide a potential bias.) A series of ‘selected quotes’ are presented, without being clear how such quotes were chosen. Whilst it’s clear that the course had a very positive impact on some respondents, there’s a risk of selection bias in the choice of quotes presented in the main paper.

**Response: **The only “selection” of the quotes was based on whether the respondent did or did not give permission for the publication of their de-identified comment. The table and spreadsheet uploaded to Zenodo contains all of the responses that the respondents agreed to allow to be published. The following sentence has now been added under “Data analysis” describing the compilation of the statements. All free text statements added by respondents who agreed to allow their responses to be published were compiled and are reported in Table 4. The discussion of the data generated acknowledge the positive results gained and some of the limitations of the study.

**Reviewer’s comment: **It would perhaps also be useful to set the discussion of this course a bit more in the overall offering of the School, and the importance it attaches to humanities-based options. Students who took this module may have also been influenced in how they viewed this particular module by participation in other courses, e.g. in narrative medicine. It’s too late to explore whether there were any mutual interdependencies/reinforcements between courses in the survey, but briefly discussing the School’s overall humanities offerings and attitudes to them might add value.

**Response: ** We have now added the paragraph below into the Discussion in response to Dr. Sabroe’s suggestion. We did not assess mutual interdependence/reinforcements between courses with questions in the survey, in part because we did not consider this possibility, but also because we wanted to keep survey as short and simple as possible so as to increase the response rate while trying to obtain the most information. The Icahn School of Medicine has been a pioneer in integrating Humanities in Medicine through a rich set of offerings to medical students. The school also created a “Humanities and Medicine” admissions process under Dean Nathan Kase’s leadership in 1987 to better enable students with a strong interest in the humanities to become physicians. Currently, the humanities offerings are under the auspices of the Academy for Medicine & the Humanities. In addition to the Pulse of Art, the Academy offers a number of courses in creative writing, music, history, the social sciences, and the visual arts. The Academy also sponsors mentored research opportunities, which include students creating courses for other students. The school also allows students to fulfill their research graduation requirement by participating in a Medical Humanities Scholars Track.

**Reviewer’s comment: **A minor point, but in the supplemental materials, one link (reading and misreading faces) needs a proper link direct to the source material.

**Response: **Thank you for letting us know. We have fixed the link.

 As a point of discussion, the final paragraph of the discussion causes me to wonder how we present the importance and benefit of such courses.

**Reviewer’s comment: **I cannot conceive of a future in which the practice of medicine will be easy. It will always be challenging, acutely and over a lifetime. I am all in favour of all wellness programmes. I support approaches that can positively impact on the lifetime stress of our career, as well as those that offer diverse ways of learning about illness. I think we should be incorporating humanities-based learning, knowledge and wisdom into training and practice as part of a standard to support clinician learning, practice, identity formation and wellbeing, for the benefits of our patients, societies, and ourselves. But… I believe the work of addressing clinician distress and burn-out requires deep reforms of working practice, clinician support etc, and that the work of creating more sustainable, survivable careers is to some degree an independent (and great) problem.

**Response: **We share Dr. Sabroe’s view that clinical distresses and burn-out requires structural reforms of many aspects of medical training and practice, and that much of this is independent of courses like ours. At the same time, even though we did not explicitly provide questions about burn-out or wellness, several spontaneous comments indicated that at least for some of the respondents, the time in our course provided an antidote to the stresses of their medical responsibilities, and we were struck by similar comments about humanities courses in the literature. Thus, since we recognize the need for a holistic approach to physician well-being, we think it worth exploring how courses like ours can contribute to that goal.

# Reviewer report: Reviewer response for version 1

**DOI:** 10.21956/mep.21537.r38117

**Published:** 2024-08-07

**Authors:** Vincenza Ferrara

**Affiliations:** 1 Sapienza University of Rome, Rome, Italy

It is an interesting study. It would be interesting to know if at the end of the courses a qualitative or quantitative questionnaire was administered regarding the impact on observation skills, empathy or well-being. It would also be interesting to know how the artworks with which to activate the discussion of participant are chosen and what type of method is applied both in the classroom and in the museum. I confirm that the paper is very important in the study of Humanities in Medical Education and that is of an acceptable scientific standard with minor revisions for a indexed.

Is the case presented with sufficient detail to be useful for teaching or other practitioners?

Partly

Is the work clearly and accurately presented and does it cite the current literature?

Partly

If applicable, is the statistical analysis and its interpretation appropriate?

Yes

Is the case’s background and context in sufficient detail?

Yes

Are all the source data underlying the results available to ensure full reproducibility?

Yes

Are the conclusions drawn adequately supported by the results?

Yes

Reviewer Expertise:

My area of ​​expertice and research is the application of the Visual Thinking Strategies method and other artistic practices for the training of medical and nursing students and continuing education in the healthcare area for developing skills and limiting stressof personell

I confirm that I have read this submission and believe that I have an appropriate level of expertise to confirm that it is of an acceptable scientific standard, however I have significant reservations, as outlined above.

# Response

**Published:** 2024-08-16

**Authors:** Barry Coller

**Affiliations:** Laboratory of Blood and Vascular Biology, Rockefeller University, New York, NY, USA

We thank Dr. Ferrara for the careful review, and we are pleased that he judges our study to be interesting and very important in the study of Humanities in Medical Education. We offer the following response to his comments. It is an interesting study.

**Reviewer’s comment:** It would be interesting to know if at the end of the courses a qualitative or quantitative questionnaire was administered regarding the impact on observation skills, empathy or well-being.

**Response:** After each session of the course, we request that students provide responses to a series of questions related to that session, but we also encourage general comments and suggestions. In addition, at the end of the course, we request that students complete a survey related to the course as a whole. In the latter, we ask the following questions. “With a scoring system of 1-5, with 5 being the best,

 how would you rate the success of the course in achieving each of the following goals:

1. Encourage careful observation,
2. Provide an understanding of some of the key milestones in medical history,
3. Encourage awareness of, and sensitivity to, the impact of illness on patients and their families.

**Reviewer’s comment:** ” We do not specifically use the term empathy, but we think that question 3 relates to empathy, and we prefer to avoid the term empathy itself because of the heavy emotional overlay associated with this word. We have not yet asked about well-being, but in response to the reviewer’s question, we will add a question related to well-being.

**Response:** It would also be interesting to know how the artworks with which to activate the discussion of participant are chosen and what type of method is applied both in the classroom and in the museum.

 The art works are selected primarily based on both their art historical and medical historical significance, either directly or indirectly. These representational works are supplemented with other works of art to demonstrate the power and variety of art, including examples of abstraction, photography, and sculpture that reinforce the key theme of the session. In the classroom, the emphasis is encouraging the students to observe the works in silence for several minutes and then begin to discuss what they observed and their reaction to the work as a whole, in the context of the topic. This approach incorporates key elements of Visual Thinking Strategies.

 The Guggenheim visits are led by a highly respected museum educator, Sharon Vatsky, who has extensive experience in developing and leading educational activities. She has written two books on this topic, *Museum Gallery Activities: A Handbook (American Alliance of Museums)* and *Interactive Museum Tours: A Guide to In-Person and Virtual Experiences*. She engages the students in first observing and then using a series of exercises to elicit their responses, including passing papers around from person to person, in which each person in turn writes their response to a series of questions, followed by a group read-back.

 The New York Academy of Medicine Rare Book Room Librarian, Arlene Shaner, displays a series of books that relate to the course or to the individual interests of the students, providing a historical context and comparing the works to others published at the same time. She emphasizes how the works also fit into the history of book printing and publishing, and figure production.
