# Supplementary material for: Long-Term Impact of a Medical School Course on the Intersection of Art and Medical History
Source: MedEdPublish (2016). 2024 Jul 9;14:51. [Version 1] doi: 10.12688/mep.20112.1 (PMC11362722; doi:10.12688/mep.20112.1)
Supplement: Peer review history

# Response

**Published:** 2024-08-16

**Authors:** Barry Coller

**Affiliations:** Laboratory of Blood and Vascular Biology, Rockefeller University, New York, NY, USA

We thank Dr. Swain for the careful review and appreciate her identifying the need for data on the longitudinal impact of medical humanities as a strength of our manuscript. I have a few comments/suggestions below for the authors:

1. **Reviewer’s Comment:** I recommend an editor review the piece. There are many run-on sentences that make sections hard to read.
**Response: **We would be pleased to review any sentences that Dr. Swain or an editor thinks needs revision.
2. **Reviewer’s Comment: **The very first sentence is misleading. Medical humanities education is not a recent trend, it has been used in US medical education for 20+ years and much longer in European medical schools. I suggest this sentence is adjusted to reflect this fact.    
                                                                                                        
**Response: **We thank Dr. Swain for the suggestion. We have modified the sentence accordingly. It now reads as indicated below.   Calls for integration of the humanities into medical education go back at least to the 1940s, but judging by PubMed citations, there has been a dramatic increase in interest and scholarship in this area during the past two decades.
3. **Reviewer’s Comment: ** I think the questionnaire to alumni would have benefited from a Likert scale. The yes/no questions limit the utility of the information collected. I realize this data has already been collected, but in a methods section it would be helpful to discuss why they decided on yes/no vs something more detailed. I think this deficit could have been made up for by a content analysis of the answers (if there had been more of them).
**Response: **Dr. Swain makes a good point. In reflecting on our choice of yes/no answers, we believe it derived from our sense that most of the answers to the questions would be binary, such as whether they still think about the course. We acknowledge that some of the others may have lent themselves to Likert scale assessment and will consider this for future research.  
4. **Reviewer’s Comment: **If the authors are going to comment on burnout it makes more sense to do so earlier in the article and then circle back to it in the discussion section.
**Response: **We thank the reviewer for the suggestion, and we have now added the sentence below in the Introduction. Although not one of our stated goals, in the course of our analysis we noted that a number of respondent comments touched on the issue of physician burnout and wellness, a subject that has been a focus of recent attention, and so we briefly discuss the potential of courses like ours to contribute to physician wellness.
5. **Reviewer’s Comment: **Curious if the authors plan to use any of the data collected for this article to make changes to their current course.
**Response: **Based on the comments related to wellness, we are thinking about how to modify our course or create a related course that focuses more on this important issue. We now plan to ask specifically about wellness in our course survey.
